# Perceived stress of adolescents during the COVID-19 lockdown: Bayesian multilevel modeling of the Czech HBSC lockdown survey

**DOI:** 10.3389/fpsyg.2022.964313

**Published:** 2022-09-29

**Authors:** Jana Furstova, Natalia Kascakova, Dagmar Sigmundova, Radka Zidkova, Peter Tavel, Petr Badura

**Affiliations:** ^1^Olomouc University Social Health Institute, Palacky University Olomouc, Olomouc, Czechia; ^2^Psychiatric-Psychotherapeutic Outpatient Clinic, Pro Mente Sana, Bratislava, Slovakia; ^3^Faculty of Physical Culture, Palacky University Olomouc, Olomouc, Czechia

**Keywords:** perceived stress, COVID-19 lockdown, HBSC, adolescent, Bayesian multilevel regression

## Abstract

**Objective:**

Long-term isolation, including lockdowns and quarantines, may have a distressing effect on anyone experiencing it. Adolescent brain architecture is very sensitive to environmental adversities, and the mental health development of adolescents may be particularly vulnerable during the pandemic era. In order to better understand the triggers for perceived adolescent stress (PSS) during the COVID-19 lockdown, the present study aimed to assess the effects of social well-being and changes in time use during the lockdown, as well as the family COVID experience of adolescents.

**Methods:**

The sample for this study comprised *n* = 3,440 adolescents (54.2% girls; mean age = 13.5 ± 1.6 years). Bayesian correlations between PSS, health and well-being variables were assessed. PSS was then modeled as an outcome variable in a series of nested Bayesian multilevel regression models.

**Results:**

The negative impact of the COVID-19 lockdown was more apparent in girls. PSS was moderately correlated with adolescent health and well-being. The strongest predictor of higher level of PSS was frequent feeling of loneliness. On the contrary, lower level of PSS was most associated with having someone to talk to.

**Conclusion:**

Long-term social isolation of adolescents could be harmful to their mental health. Psychological coping strategies to prevent the consequences of social isolation and development of mental health problems should be promoted on the individual, family, and even community level.

## Introduction

In March 2020, the World Health Organization (WHO) declared a pandemic to contain the spread of COVID-19 (Mahase, 2020). Subsequently, countries around the world adopted lockdowns, social distancing, and school closures complemented with online education. Restrictive measures were gradually implemented to prevent gatherings of people and reduce the incidence of the infection (Panchal et al., 2021). Thus, the pandemic became a risk not only for physical health, it started affecting mental health, as well (Pfefferbaum and North, 2020). Indeed, long-term isolation, including lockdowns and quarantines, may have a distressing effect (Brooks et al., 2020; Racine et al., 2022). For adolescents in particular, the adverse effects of pandemic lockdowns on mental health are substantial (Kiss et al., 2022). According to some authors, the psychological consequences of the pandemic may even outlast its physical impact (Brooks et al., 2020).

Adolescent brain architecture is very sensitive to environmental adversities (Eiland and Romeo, 2013; Fuhrmann et al., 2015), and the mental health development of adolescents may be particularly vulnerable in the pandemic era (Panchal et al., 2021). During the lockdown, adolescents were exposed to chronic stress conditions, such as forced isolation from peers due to school closures, social distancing, restrictions in leisure-time activities, loss of a sense of security and safety, and fears about the future (Gruber et al., 2021; Panchal et al., 2021). Frequently reported psychosocial outcomes of the COVID-19 lockdowns on adolescents were sleep disruption, loneliness, anger, irritability, or a worsening of preexisting psychiatric symptoms (Panchal et al., 2021; Porter et al., 2021). Globally, adolescents have been reporting higher rates of depression, anxiety, and overall mental health deterioration due to the pandemic (Panchal et al., 2021; Kiss et al., 2022). The adolescent suicide rate has also been increasing (Hoekstra, 2020; Mayne et al., 2021). Girls (Magson et al., 2021; Kiss et al., 2022) and older adolescents (Chen et al., 2020; Panchal et al., 2021) were reported to be at higher risk of poor mental health during the COVID-19 pandemic. Even before the pandemic, adolescent girls were shown to suffer from a greater psychological distress accompanied with higher level of physical and mental health complaints than boys (Eiser et al., 1995; Wade et al., 2002). A steady trend of increased gender differences in health complaints with higher adolescent age has been indicated in many Western countries (Torsheim et al., 2006). The excess reporting of psychosomatic symptoms in female adolescents tends to be related to self-esteem and body image (Sweeting et al., 2007), as well as differing rates of biological development, societal expectations, lifestyle, and behaviors (Sweeting, 1995).

According to previous studies, decrease in physical activity and increase in time spent being sedentary, especially in front of a screen and using technology/social media belong among the most common changes found in the behavior of adolescents during periods of lockdown (Jones E. et al., 2021; Panchal et al., 2021). All the above have previously been identified as risk factors for developing mental health problems (Hoare et al., 2016). Prior research has also shown that poor mental health in adolescents was influenced by distress, frustration or even anger in their parents, which could have resulted in overreactions and increased family violence (Cluver et al., 2020; Achterberg et al., 2021). External stressors thus elicit family tension, which leads to a decline in well-being in both parents and children (Achterberg et al., 2021). However, all types of routine, social support, family communication and appropriate leisure-time activities seem to have had a positive impact on adolescent mental health during the pandemic (Donker et al., 2021; Panchal et al., 2021). On the other hand, the decrease in social and school obligations may have reduced the pressure on both children and parents; thus, some families could have experienced the pandemic as a form of relief (Hoekstra, 2020; Bruining et al., 2021).

The level of perceived stress characterizes the extent to which a person views his/her life as being unpredictable, uncontrollable, and stressful (Cohen et al., 1983). It is seen as a psychological risk factor with an impact on the outbreak and development of mental disorders among adolescents (Lindholdt et al., 2021). Half of the mental disorders, including depression, anxiety disorders, suicidality, etc., emerge or worsen during adolescence (Paus et al., 2008; Gruber et al., 2021), and their consequences persist into adulthood (Aneshensel, 1992). Psychological stress during adolescence has also been identified as a risk factor for alcohol and drug use, with the consequent perils of developing addiction (Aseltine and Gore, 2000; Sinha, 2008). Further, adolescent stress is associated with obesity and abnormalities in immune, metabolic and cardiovascular functions, with greater risk for subclinical atherosclerosis in adulthood (De Vriendt et al., 2009; Low et al., 2009; Pervanidou and Chrousos, 2012). Increased levels of psychological stress during adolescence may be especially harmful given that coping skills, psychosocial and physiological stress response systems develop during this period (Repetti et al., 2002; Low et al., 2009; Gavin et al., 2020). The perceived stress of adolescents thus deserves attention in COVID-19 research, due to its enduring impact on physical and mental health, as well as on overall well-being (Jones E. et al., 2021).

The severity of pandemic preventive measures varied per countries, just as the outcomes may vary. The challenges of the pandemic, however, have been scattered around the world, and its consequences need to be monitored. During the first national lockdown in the spring of 2020, the Czech Republic was among the very strict countries in terms of preventive measures relative to the number of infections (Stringency Index, 2020). There is, however, still little known about its effect on Czech adolescents’ perceived level of stress and their well-being. This study aims to (1) assess gender differences in the COVID-19 lockdown experience of Czech adolescents, (2) evaluate the correlations between adolescents’ perceived level of stress and their well-being, and (3) estimate the associations between potential pandemic stressors and the perceived level of stress among adolescents during the spring 2020 lockdown.

## Materials and methods

### Data and participants

Data for this study is connected to the Health Behavior in School-aged Children (HBSC) study,^1^ which is an international cross-sectional survey on adolescent health. There is a standardized international protocol for the data collection process among 11-, 13- and 15-year-old school children (Inchley et al., 2018). During the first Czech COVID-19 lockdown in the spring of 2020, schools were asked to participate in an unscheduled round of data collection with the HBSC survey questionnaire adapted to capture the impact of the lockdown on the adolescent health (Cosma et al., 2021). Out of 234 randomly selected schools from all 14 Czech administrative regions, 144 schools were willing to participate in the survey. Data was collected online during June 2020. The final sample for this study contains *n* = 3,440 participants from the 5th, 7th, and 9th grades (54.2% girls, mean age = 13.5 ± 1.6 years). Participation in the survey was voluntary and anonymous, with informed consent from the participants’ parents. The participants were not paid and no other incentives were provided for their participation. The research was conducted according to the guidelines of the Declaration of Helsinki and was approved by the ethics committee of the Faculty of Physical Culture, Palacky University Olomouc (No. 65/2020).

### Missing data

Missing data on item level totaled 0% to 6.4% (1.8% missing values on average). We assumed that the data is MCAR (Missing Completely At Random) and performed all the analyses twice; first, with complete cases only (with case-wise deletion of missing values), and second, with all missing data imputed using a Bayesian imputation process in the Rethinking package in R software (McElreath, 2020). As the results with complete cases were analogous to those from the imputed data, in this study we report the results based on the imputed dataset only. In Bayesian imputation, the measured values provide priors for the missing values. The priors are then updated by the relationship between the predictors and the outcome used in the imputation. Each missing value is then replaced by the maximum *a posteriori* probability (MAP) estimate.

### Variables and measures

#### Sociodemographic variables

The respondents reported their age, gender (boy–girl) and the region where they go to school (one of the 14 Czech administrative regions).

#### Health and well-being

The short form of the *Perceived Stress Scale* (PSS) was used to assess the respondents’ level of perceived stress. The instrument is a brief 4-item scale measuring stress perception during the last month (Cohen, 1988). Responses are assigned on a five-point Likert scale (0 – never, to 4 – very often), with the items summed to provide a total PSS score ranging from 0 – least stressed, to 16 – most stressed. In international validation studies, the internal consistency of the PSS-4 scale ranged from *α* = 0.55 (Lee et al., 2015) to *α* = 0.77 (Warttig et al., 2013). In a Czech adult sample, the internal consistency of the PSS-4 was *α* = 0.83, *ω* = 0.80 (Figalova and Charvat, 2021). *Life satisfaction* captured how adolescents felt about their life at present. It was measured using the Cantril ladder, which is a visual scale with 11 possible values ranging from 0 – worst possible life, to 10 – best possible life (Levin and Currie, 2014). The ladder was shown to be understood by adolescents and being reliable and valid measure in this age group (Levin and Currie, 2014). *Psychological and somatic complaints* were assessed using the HBSC Symptom Checklist (Haugland and Wold, 2001). Respondents reported the frequency of psychological symptoms (i.e., feeling low, irritable or in a bad mood, nervous and having difficulties falling asleep) and somatic symptoms (i.e., headache, stomachache, backache, and feeling dizzy) during the past month. A five-point scale is used for rating the two subscales, from 0 – about every day, to 4 – rarely or never. A summary score with values from 0 to 16 in each subscale is assessed, with higher scores representing more frequent complaints (*α* = 0.61 and 0.77 for somatic and psychological subscale, respectively, in the present sample). The checklist was shown to have good discriminant and internal validity (Gariepy et al., 2016) and test–retest reliability (Haugland and Wold, 2001). *Self-rated health* was reported on a four-point scale from 1 – poor, to 4 – excellent using a question adapted from the study of Kaplan and Camacho (1983). It was described as a valid measure for large-scale population-based surveys in adolescents and young adults by Allen et al. (2016), who found it was an equivalent construct across various population groups.

#### Social well-being during the COVID-19 lockdown

The respondents were asked to report how often during the spring 2020 lockdown they felt (1) lonely, (2) felt being part of a group of friends, and (3) had people they could talk to about important things. The responses ranged from 1 – never, to 5 – very often, to each question. The items were adapted for adolescent use from the original R-UCLA scale (Hughes et al., 2004).

#### Changes in time use during the COVID-19 lockdown (compared to pre-lockdown)

The adolescents were asked to report whether they spent less or more time on several activities during the spring 2020 lockdown than before the lockdown. The compared activities were (1) schoolwork, (2) leisure time, (3) sleep on weekdays, and (4) sleep on weekends. The responses ranged from 1 – definitely less, to 5 – definitely more, to each question. The questions were developed *ad hoc* to study the perceptions of changes during the first wave of lockdown. The adolescents were also asked “Over the past 7 days, on how many days were you physically active for a total of at least 60 min per day?” The answers ranged from 0 to 7 days. The question was developed by Prochaska et al. (2001), validated against accelerometers, and manifested good test–retest reliability.

#### Family COVID-19 experience

The Perceived Impact of the 2008/09 Economic Crisis Scale (Due et al., 2019) was adapted for the lockdown situation to assess household disruptions due to the COVID-19 pandemic. There were two items assessing economic disruptions – one or both parents losing their jobs, having less money in the household; three items assessing psychosocial disruptions – felt cramped in the house, more family disputes, more parental stress; two items assessing opportunities – learn new things, more time to engage in joint activities with family that they all enjoyed. The responses were coded 1 – yes, 0 – do not know, and-1 – no. The respondents were also asked whether they or their family members were diagnosed with COVID-19. There were only 21 positively diagnosed respondents in the sample (0.6%), and 47 positively diagnosed members of their families (1.4%). These numbers were so negligible that we did not include the variables in the analyses.

### Statistical analyses

All analyses were performed using the R 4.1.2 statistical software. Bayesian methods for all statistical analyses were used. Highest density intervals (HDIs) are presented together with the posterior probability distribution estimates. HDIs summarize the most credible values of the posterior distributions. Their span in this study was set to 95%.

For a comparison of the descriptive characteristics between males and females, the Bayesian *t*-test and Bayesian proportion test was used, implementing the Bayesian First Aid package (Baath, 2014). The Bayesian First Aid package was also used for assessing the Bayesian correlations between the PSS, health and well-being variables. Weakly informative and uninformative default priors, as described in Kruschke (2013), were used for these analyses: normal prior distributions with a large standard deviation for the population means, uniform priors for standard deviations and a shifted-exponential prior for the normality parameter. The Bayesian Monte Carlo Markov Chain (MCMC) process in JAGS was used for estimating the posterior probability distributions (Plummer, 2003).

Three nested varying-intercepts multilevel regression models were then fitted using the administrative regions of the Czech Republic as the higher level and individual respondents as the lower level. Model 1 estimated the effect of social well-being during the COVID-19 lockdown. In Model 2, the effect of social well-being and changes in time-use during the COVID-19 lockdown was assessed, while the full model (Model 3) further evaluated the effect of the family COVID-19 experience in the respondents’ families. All three models were adjusted for gender and age of the respondents. In the regression models, standardized variables (*z*-scores) were used to allow for a comparison of the variables of different scales. Only the variables representing the family COVID-19 experience of the respondents did not have to be standardized, given their natural categorization (−1, 0, 1 values). Weakly informative priors were used to regularize the estimates. Hyperparameters *α* ~ Normal (0,1) and *σ* ~ Cauchy (0,2.5) were used for estimating the random effects with the hyperprior Normal (*α*, *σ*). Given the standardization of the variables, the Normal (0,1) prior was employed for estimating the fixed effects in the regression models. Using the Normal (0,1) prior states it is likely for the estimates to have a majority of the values within one standard deviation from the mean. The Rethinking package (McElreath, 2020) was used for multilevel regression modeling. The MCMC sampling was performed with the Stan sampler (RStan, 2021), using 10,000 iterations in 4 chains. The performance of the models was compared using the Watanabe–Akaike information criterion (WAIC), which evaluates the predictive accuracy for the fitted Bayesian models (Gelman et al., 2013). A lower value of the WAIC suggests a better fit. The analytical script for the R software is available as a Supplementary File.

## Results

### Descriptive characteristics

Descriptive characteristics of the dataset (*n* = 3,440; mean age = 13.45 years, SD = 1.62) are presented in Table 1. There were 1,866 girls (54.2%) and 1,574 boys (45.8%). In general, girls reported higher levels of perceived stress, lower life satisfaction and poorer self-rated health, including higher levels of psychological and somatic complaints. Girls also felt more lonely. No meaningful difference was found between boys and girls in the frequency of feeling to be a part of a group of friends or having the opportunity to talk to someone (the HDI intervals contain 0). Also, no gender differences were found in the majority of time-use variables during the lockdown, i.e., changes in sleep time, time spent doing schoolwork or amount of leisure time. However, girls reported being more physically active during the lockdown than boys; on average, girls were physically active for at least 60 min per day more than 4 days during their past week. The proportion of girls reporting negative family disruptions due to COVID-19 was higher than that of boys (25% of girls felt cramped at home; almost 25% of girls reported higher frequency of disputes at home, compared with the rates for boys – 14% and 17%, respectively). Over 40% of the adolescents (both genders) reported their parents being more worried than before. On the other hand, the majority of the adolescents saw the lockdown as an opportunity to learn new things (68% of girls and 60% of boys) and had more time for enjoyable joint family activities (56% of girls and 62% of boys).

**Table 1:** Descriptive characteristics of the sample.

Variable	Total *n* = 3,440	Girls *n* = 1866	Boys *n* = 1,574	Mean posterior group difference^a^	95% HDI credible interval
	Mean (SD)	Mean (SD)	Mean (SD)		
Age	13.45 (1.62)	13.48 (1.62)	13.41 (1.63)	−0.07	−0.18, 0.04
**Health and well-being**					
Perceived stress (score 0–16)	6.31 (2.78)	6.65 (2.90)	5.90 (2.57)	−0.74	−0.92, −0.55
Life satisfaction (score 1–10)	7.73 (1.78)	7.53 (1.86)	7.97 (1.65)	0.44	0.33, 0.56
Psychological complaints (score 0–16)	4.57 (3.88)	5.11 (4.05)	3.93 (3.57)	−1.20	−1.46, −0.95
Somatic complaints (score 0–16)	1.96 (2.44)	2.31 (2.64)	1.54 (2.12)	−0.65	−0.77, −0.53
Self-rated health (score 1–4)	3.27 (0.66)	3.22 (0.66)	3.33 (0.65)	0.11	0.06, 0.15
**Social well-being during COVID-19 lockdown**					
Lonely (score 1–5)	2.18 (1.19)	2.42 (1.22)	1.90 (1.09)	−0.52	−0.60, −0.45
Part of group of friends (score 1–5)	2.95 (1.28)	2.92 (1.23)	3.00 (1.33)	0.08	−0.01, 0.17
Could talk to someone (score 1–5)	3.59 (1.22)	3.61 (1.20)	3.56 (1.25)	−0.05	−0.13, 0.04
**Changes in time use during COVID-19 lockdown^b^**					
Sleep time weekends (score 1–5)	3.50 (0.99)	3.50 (0.97)	3.50 (1.01)	0.00	−0.07, 0.07
Sleep time schooldays (score 1–5)	3.80 (1.06)	3.80 (1.05)	3.80 (1.08)	0.01	−0.07, 0.08
School work (score 1–5)	3.46 (1.29)	3.50 (1.26)	3.43 (1.32)	−0.07	−0.16, 0.02
Leisure time (score 1–5)	3.85 (1.15)	3.82 (1.13)	3.89 (1.18)	0.08	0.00, 0.16	Physical activity during past week (0–7 days)	3.95 (2.11)	4.08 (2.05)	3.80 (2.18)	−0.29	−0.43, −0.14	**Family COVID-19 experience^c^**	It felt cramped in my house	681 (19.80)	467 (25.03)	214 (13.60)	−0.12	−0.15, −0.10	More disputes at home than before	725 (21.08)	454 (24.33)	271 (17.22)	−0.08	−0.11, −0.05	There was less money at home	495 (14.39)	270 (14.47)	225 (14.29)	0.00	−0.03, 0.02	One or both parents lost their job	286 (8.31)	177 (9.49)	109 (6.93)	−0.03	−0.05, −0.01	My parents worried more than before	1,398 (40.64)	753 (40.35)	645 (40.98)	0.00	−0.03, 0.04	Opportunity to learn new things	2,212 (64.30)	1,866 (68.01)	943 (59.91)	−0.09	−0.12, −0.05
More time for enjoyable joint activities	2,023 (58.81)	1,047 (56.11)	976 (62.01)	0.06	0.02, 0.09

Results of the Bayesian *t*-test and the Bayesian test of proportions comparing girls and boys.

a Boys vs. girls; SD = standard deviation.

b Changes in time use compared to pre-lockdown, and physical activity.

c Proportion of positive (“yes”) responses to each question.

### Correlates and predictors of the level of perceived stress

The level of perceived stress of adolescents was moderately correlated with their well-being during the lockdown: The PSS was positively correlated with psychological and somatic complaints (median posterior *r* = 0.51, 95% HDI [0.48, 0.53]; median posterior *r* = 0.33, 95% HDI [0.29, 0.36], respectively) and negatively correlated with self-rated health (median posterior *r* = −0.32, 95% HDI [−0.35, −0.29] and life satisfaction (median posterior *r* = −0.46, 95% HDI [−0.49, −0.43]).

The results of standardized multilevel regression analyses predicting the level of adolescent perceived stress are summarized in Table 2. All three models have been adjusted for gender and age. In Model 1, the effect of social well-being during the COVID-19 lockdown on stress was analyzed. The analysis showed loneliness was associated with a higher level of stress (posterior mean *β* = 0.31, 95% HDI [0.28, 0.34]), while being a part of a group of friends or having someone to talk to was associated with a lower level of perceived stress (posterior mean *β* = −0.07, 95% HDI [−0.10, −0.04]; posterior mean *β* = −0.19, 95% HDI [−0.23, −0.16], respectively).

**Table 2:** Bayesian multilevel regression analysis of covariates predicting the level of perceived stress in adolescents during the spring 2020 lockdown.

Predictor	β (95% HDI)^a^		
	Model 1	Model 2	Model 3
Gender^b^ (female vs. male)	0.13 (0.07, 0.19)	0.14 (0.08, 0.20)	0.13 (0.06, 0.18)
Age^b^	0.05 (0.02, 0.08)	0.05 (0.02, 0.08)	0.03 (0.00, 0.06)
**Social well-being during COVID-19 lockdown** ^**c**^			
Lonely	0.31 (0.28, 0.34)	0.30 (0.27, 0.33)	0.24 (0.20, 0.27)
Part of group of friends	−0.07 (−0.10, −0.04)	−0.06 (−0.09, −0.03)	−0.05 (−0.08, −0.02)
Could talk to someone	−0.19 (−0.23, −0.16)	−0.19 (−0.22, −0.16)	−0.15 (−0.19, −0.12)
**Changes in time use during COVID-19 lockdown** ^**c**^			
Sleep time weekends		0.06 (0.03, 0.09)	0.06 (0.02, 0.09)
School work		0.02 (−0.01, 0.05)	0.03 (0.00, 0.06)
Leisure time		−0.03 (−0.06, 0.00)	−0.01 (−0.04, 0.02)
Physical activity during past week		−0.04 (−0.07, −0.01)	−0.03 (−0.06, 0.00)
Sleep time schooldays		−0.06 (−0.10, −0.03)	−0.05 (−0.09, −0.02)
**Family COVID-19 experience**			
It felt cramped in my house			0.12 (0.08, 0.16)
More disputes at home than before			0.08 (0.05, 0.12)
There was less money at home			0.07 (0.03, 0.11)
One or both parents lost their job			0.06 (0.01, 0.12)
My parents worried more than before			0.02 (−0.01, 0.06)
Opportunity to learn new things			−0.07 (−0.10, −0.03)
More time for enjoyable joint activities			−0.07 (−0.10, −0.03)
Variance for region	0.07 (0.02, 0.13)	0.08 (0.03, 0.13)	0.17 (0.10, 0.25)
WAIC	8974.5	8951.0	8805.7

a Posterior distribution mean and its 95% credible interval.

b Gender and age are used as controlling variables in all models.

c Standardized variables (z-scores) were used.

In Model 2, the effect of social well-being and changes in time use during the COVID-19 lockdown on the stress level was analyzed. The effect of social well-being remained almost the same as in Model 1. In addition to that, a higher level of perceived stress was associated with more sleep on the weekends and less sleep on school days (posterior mean *β* = 0.06, 95% HDI [0.03, 0.09]; posterior mean *β* = −0.06, 95% HDI [−0.10, −0.03], respectively) compared to the time before the lockdown. On the other hand, more days spent with physical activity lowered the level of stress (posterior mean *β* = −0.04, 95% HDI [−0.07, −0.01]). There was no substantial effect of the perceived changes in the amount of schoolwork and leisure time during the period of school closures due to the lockdown.

In Model 3, the effect of social well-being and changes in time use, together with the COVID-19 experience in the family, was analyzed. The effect of social well-being and changes in time use were analogous to those in Model 2. The negative family disruptions due to COVID-19, i.e., feeling cramped at home, having more disputes, parents losing jobs or less money at home, were associated with a higher level of perceived stress. The strongest effect of family disruptions was found in feeling cramped (posterior mean *β* = 0.12, 95% HDI [0.08, 0.16]). Both positive impacts of the lockdown, i.e., having the opportunity to learn new things and having more time for enjoyable joint family activities, lowered the level of perceived stress (posterior mean *β* = −0.07, 95% HDI [−0.10, −0.03]).

**Figure 1:**
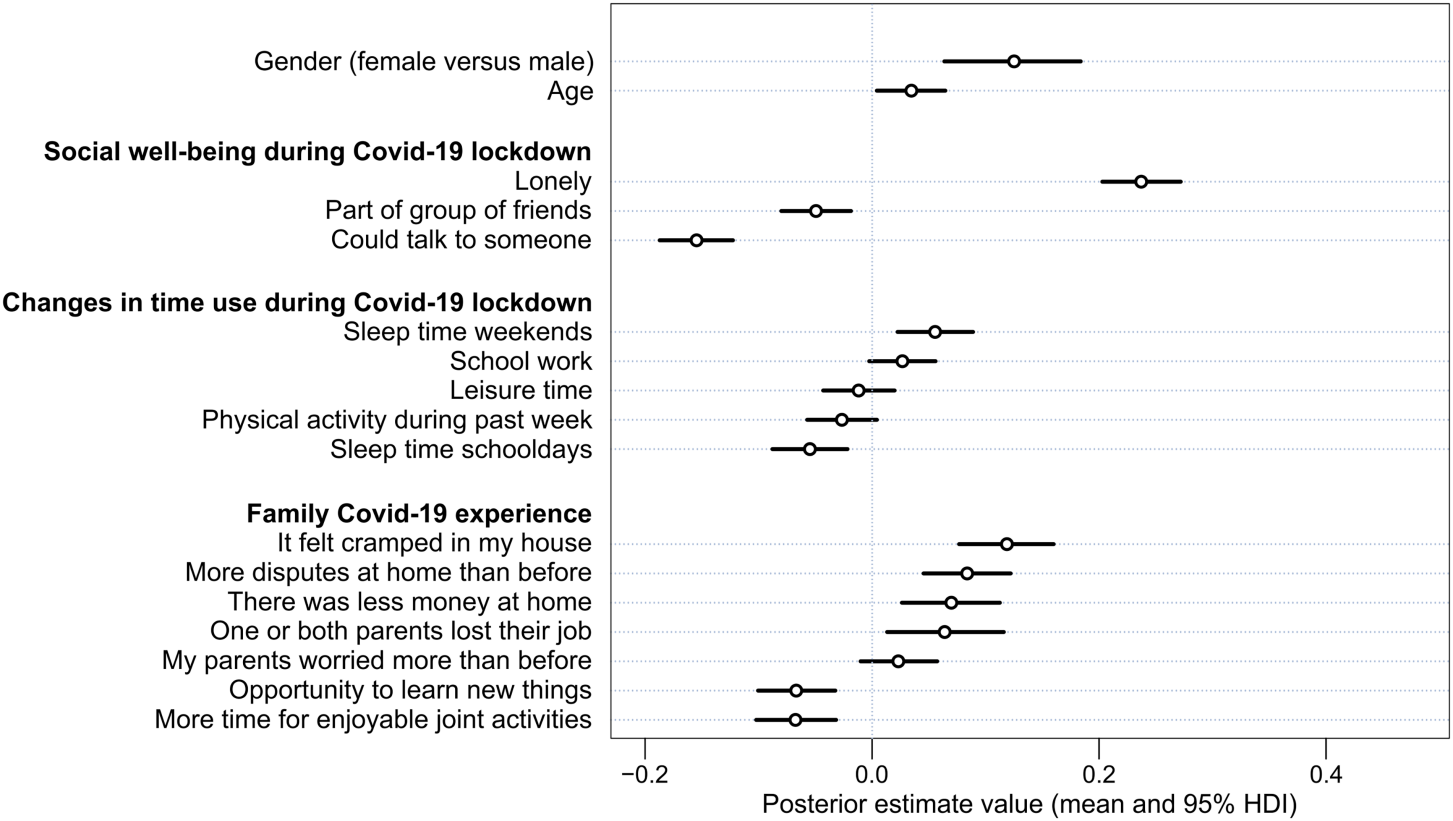
Posterior estimate mean values and uncertainty (95% HDI intervals) of stressors in adolescents during the spring 2020 lockdown (Model 3). Standardized variables (*z*-scores) were used.

From the best fitting Model 3 (considering its WAIC value) it can be concluded that the social well-being of adolescents (i.e., loneliness and not having someone to talk to) remained the strongest set of predictors of higher level of perceived stress among adolescents even after controlling for the changes in time use, and their family COVID-19 experience. A graphical interpretation of the posterior estimates of Model 3 is available in Figure 1.

## Discussion

This study aimed to assess gender differences in the COVID-19 lockdown experience of Czech adolescents, to evaluate the correlations between adolescent perceived level of stress and their well-being, and to find predictors of the perceived level of stress. The analyses suggest that the negative impact of the COVID-19 lockdown was more apparent in girls and the perceived level of stress was moderately correlated with adolescents’ health and well-being. The strongest predictor of higher level of PSS was frequent feeling of loneliness. On the contrary, lower level of PSS was most associated with having someone to talk to.

### Perceived stress, health, and well-being

Our findings support those of others, who found that perceived stress in adolescents was negatively related to health (Gruber et al., 2021; Panchal et al., 2021; Kiss et al., 2022), well-being and life satisfaction (Porter et al., 2021). Adolescence is a sensitive period in life, with extensive physical and cognitive changes and development in social and emotional areas (Steinberg, 2005). These changes involve transformation of emotional and social support from parents to friends, leading to a greater autonomy of the young person (Berndt, 2002). Moreover, the development of adolescents’ personality and identity is directly connected to their coexistence with peers (Brechwald and Prinstein, 2011). The preventive measures adopted during the pandemic to stop the virus from spreading came rapidly and with the pronounced distress of the whole society. Young people are usually more flexible and compliant to accepting new situations and changes (Cheng et al., 2014). They can, however, also be more vulnerable to abrupt changes in their lives because of the lack of psychological capabilities of resilience and coping that have not yet had time to develop (Romeo, 2013). The mental health effects of the pandemic on adolescents have been previously reported, particularly in girls (Branje and Morris, 2021; Chawla et al., 2021; Magson et al., 2021), who are systematically reported to be at higher risk of emotional problems and subsequent mental health problems (Kuehner, 2017; Kiss et al., 2022). On the other hand, the reporting might be skewed by the differing social expectations, lifestyles, and behaviors for girls and boys (Gadin and Hammarstrom, 2005; Maclean et al., 2010). In European countries, boys are more often than girls reminded that they are supposed to be strong and in control, including their physical and mental health (Maclean et al., 2010). Before the pandemic, girls were systematically reported to be less physically active than boys (Slater and Tiggemann, 2011; Telford et al., 2016). Physical activity is considered one of the most important factors for maintaining health and well-being (Penedo and Dahn, 2005). During the pandemic, physical activity level of adolescents declined worldwide regardless of gender (Gobbi et al., 2020; Roe et al., 2021; Dana et al., 2022) and thus negatively affected their physical and mental health (Bates et al., 2020). Some studies have shown, however, that adolescent moderate and vigorous physical activities decreased while light physical activities remained the same (Sekulic et al., 2020; Tulchin-Francis et al., 2021). Pre-pandemic, boys were more engaged in organized sports and moderate-to-vigorous physical activities than girls (Nader et al., 2008; Sekulic et al., 2020). This may be connected to higher drop in boys’ activity during the pandemic lockdown with girls becoming more active than boys because of proceeding with lighter physical activities (Sekulic et al., 2020). That could also be the case in the present study where girls reported more days spent with physical activity than boys. Several previous studies have found a connection between worse mental health and higher adolescent age (Chen et al., 2020; Panchal et al., 2021; Ren et al., 2021b). In the present study, the association between age and perceived stress was not strong.

### Social well-being during COVID-19 lockdown

The social well-being of Czech adolescents (i.e., loneliness, being a part of a group of friends and having someone to talk to) was found to be the strongest set of predictors of the level of stress. Loneliness increased perceived stress, while being a part of a group of friends and having someone to talk to reduced the stress level. Loneliness is an unpleasant emotional experience when there is a contradiction between the desired and the available social contact (Loades et al., 2020). Experiencing it seems to be especially difficult for young people (Heinrich and Gullone, 2006; Laursen and Hartl, 2013). This may be caused by the importance of being part of a peer group for forming the adolescent personality and identity as well as seeking support (Brechwald and Prinstein, 2011). The links between mental health and deficiency in social contacts were extensively explored before the COVID-19 pandemic (Wang et al., 2017); however, the full extent of the possible consequences of loneliness only came to the surface during the pandemic, with ordered lockdowns and distancing measures (Loades et al., 2020; Hoffart et al., 2022). Short-term loneliness has been previously shown to be less dangerous than long-term loneliness in terms of future mental health problems (Qualter et al., 2010; Loades et al., 2020). Any possible future lockdowns thus should not be extended beyond the necessary period. To some extent, the negative effects of the lockdowns might have been mitigated by the possibility of online connection through social media. However, the quality of online communication and relationships may not be the same as meeting face-to-face (Mesch and Talmud, 2006). Even though online interactions with friends can be a sound option for reducing loneliness during times of physical distancing (Branje and Morris, 2021), it is important to be mindful of the nature of adolescent interactions (Ellis et al., 2020). Adolescents reporting greater positive online social experiences (including emotional and informational support and belonging) endorsed lower levels of loneliness. In contrary, adolescents experiencing negative online experiences that engender such feelings as exclusion or rejection were reporting greater feelings of loneliness (Magis-Weinberg et al., 2021). Another negative effect can arise when adolescents co-ruminate on negative feelings (because of uncertainty and high levels of stress during pandemics) and thus unintentionally escalate negative feelings (Ellis et al., 2020). This paradoxical effect of increased notion of friendship quality and connection but simultaneously increased depression through co-rumination has been documented especially in girls (Rose et al., 2007).

### Changes in time use during COVID-19 lockdown

During the lockdown, Czech adolescents perceived several changes in their daily routine. On average, they reported more time spent on schoolwork and on leisure activities, as well as longer sleep both on weekdays and on weekends than before the lockdown. In the present study, the only predictor of perceived stress with non-zero probability was the change in length of sleep. While longer sleep on weekdays reduced the level of stress, longer sleep on weekends conversely increased the level of stress. This result might seem ambiguous. However, the sleep patterns of adolescents on weekdays are directly connected to their sleep patterns on weekends (Crowley et al., 2014). During non-pandemic weekdays, adolescents often sacrifice sleep time to fulfill their school, extracurricular and social obligations, and thus need to catch up on the “social jet lag” and prolong their sleep on weekends (Wittmann et al., 2006). During the lockdown, remote learning allowed adolescents to sleep longer (Gruber et al., 2021; Kiss et al., 2022); there were no out-of-home extracurricular activities and no face-to-face social contacts available. These pandemic measures allowed adolescents to use their time differently and might have prevented the accumulation of sleep debt during the week (Kiss et al., 2022). Sleep problems have been previously shown to be predictors of psychological distress in adolescents (Lovato and Gradisar, 2014; Becker et al., 2021), and, conversely, healthy sleep patterns reduce their stress and the risk of future health problems (Blake and Allen, 2020). This is in line with our results; prolonging sleep duration on weekdays (and thus eliminating the social jet lag) was associated with a decrease in the level of stress. At the same time, unhealthy sleep patterns on weekdays might have led to prolonging sleep on weekends, which was then associated with an increase in the level of stress.

### Family COVID-19 experience

Due to pandemic measures, families stayed at home for a longer time period than they are used to. In the present study, the factors elevating stress of adolescents were connected to psychosocial disruptions in families (feeling cramped and having more disputes at home than before) and to economic disruptions (one or both parents lost their jobs and having less money in the household). Family relationships have been previously reported to play an important role in the adjustment of adolescents during the pandemic (Bulow et al., 2021). Family can render support and comfort for its members. However, the pandemic has challenged the well-being of all family members, not only children, as the demands of the pandemic-related measures often seemed to surpass the parents’ capacity (Weeland et al., 2021). Besides eliciting fear of the illness, the pandemic measures affected the everyday life of families through changes in their routines as well as changes in school and work obligations (Weeland et al., 2021). In response, parents more often changed their parenting strategies and behaviors towards authoritarian parenting, more often monitored their children’s activities and decreased the autonomy of their children (Bulow et al., 2021; Cassinat et al., 2021; Ren et al., 2021a). Although these changes in parenting might be explained by the parents’ effort to protect their children (Weeland et al., 2021), they could have led to more parent–child conflicts and thus increased the level of stress in both parents and children (Park and Walton-Moss, 2012). Stress and conflict potential in families can be further escalated by a cramped housing situation, especially in times when there are few opportunities to escape their homes (Lips, 2021). Additionally, economic distress caused by the pandemic dealt another blow to family functioning. Financial hardship is a well-known stressor affecting the well-being of families even before the pandemic (Wickrama et al., 2012; Budescu and Taylor, 2013). Thus, during times of crises (like pandemics), the necessary restrictions should be implemented with special care and considerations, especially in regions with low-income families where financial difficulties and household overcrowding could be more substantial.

Besides the negative impact of the pandemic, studies have also shown some positive effects, especially in increasing the resilience and stability of some individuals and families (Donker et al., 2021; Weeland et al., 2021), particularly those who were able to use the unanticipated time spent at home to increase family bonding (Achterberg et al., 2021). Family functioning seems to have had a major impact on adolescents’ coping with the effects of the pandemic. A supportive family environment protected adolescents from excessive distress (Magson et al., 2021), whereas problematic families with negative interactions before the pandemic had more difficulties adapting to the stressful lockdown situations (Weeland et al., 2021). The pandemic could have possibly further escalated pre-pandemic family conflicts (Achterberg et al., 2021).

In the present study, there were two factors decreasing adolescent stress levels. These were items assessing opportunities – the opportunity to learn new things and having more time to engage in joint activities with family that they all enjoyed. School closures and lack of out-of-home activities might have led to less academic and social pressure for some adolescents (Hoekstra, 2020) and given them more time to engage in new activities, on their own or with their family members. Fewer peer conflicts and bullying in a school environment may also have had a positive impact on decreasing the stress level and elevating the well-being of adolescents (Hoekstra, 2020; Bruining et al., 2021). There is a great sociodemographic and psychosocial variability of individuals and even greater variability in the way they response to the sudden changes the pandemic brought to their lives (Achterberg et al., 2021). It is important to comprehend which adolescents may rely on their resilience; they will be able to bounce back to normal, having learned and maybe even benefitted from the pandemic (Branje and Morris, 2021). On the other hand, those adolescents who are at risk of experiencing chronic negative consequences of pandemic stress should get support from their surroundings (Weeland et al., 2021). This is especially important with respect to the possible long-term impact on the adolescents’ mental health (Weeland et al., 2021; Kiss et al., 2022).

### International COVID-19 adolescent experience

The severity of pandemic preventive measures varied among countries, however, their negative consequences on mental health of adolescents have been reported across the world. In European countries such as Italy, Germany, Croatia, United Kingdom or Ireland, adolescents have experienced increase in their emotional problems, depression and anxiety connected to feelings of social isolation (Francisco et al., 2020; Forte et al., 2021; Hu and Qian, 2021; O'Sullivan et al., 2021; Ravens-Sieberer et al., 2022). In Italy, Croatia, and Romania, Forte et al. (2021) reported that lockdown significantly decreased quality of life, optimism and happiness, and increased perceived stress in adolescents. Moreover, those living in a small apartment without a prospect of going out experienced intensified feelings of sadness, anger and anxiety (Forte et al., 2021). That is in line with a study from Spain, Portugal, and Italy, where Francisco et al. (2020) found that a possibility of going outdoor (terrace or garden) decreased the level of psychological stress of adolescents. In the United Kingdom, Italy, Romania, and Croatia, boys were less likely than girls to experience emotional problems (Forte et al., 2021; Hu and Qian, 2021). The negative impact on mental health was apparent especially among adolescents in low-income, one-parent, and single-child households (Hu and Qian, 2021; Ravens-Sieberer et al., 2022). All these findings are comparable to the outcomes of the present study on a sample of Central European adolescents showing that unfavorable family conditions, stay at home orders and social distancing were related to elevated levels of stress.

Outside Europe, high rates of adolescent distress due to the pandemic disruptions of every-day life was reported in the United States (Hoffmann and Duffy, 2021) as well as in Canada (Thomson et al., 2021). These countries deal with similar post-lockdown problems in adolescents as the European countries. However, there are countries with socioeconomically disadvantaged regions (e.g., in India, Bangladesh, Jordan, Peru, Ethiopia, or Uganda), where school closures severely disrupted lives of young people in plentiful aspects beyond higher level of stress and mental health concerns (Patra and Patro, 2020; Jones N. et al., 2021; Favara et al., 2022; Nuwematsiko et al., 2022). After the schools were closed, numerous adolescents started working to provide for their families, with only a small chance of returning to school ever again (Patra and Patro, 2020; Jones N. et al., 2021; Favara et al., 2022; Nuwematsiko et al., 2022). The pandemic thus irreversibly changed their lives and their prospects of better future.

### Limitations

There are several limitations of this study: First, its cross-sectional design does not allow us to reach conclusions on causality, and it has limited longitudinal evidence. As the national lockdowns came abruptly in the spring of 2020, the possibility of planning a longitudinal study design was limited. There was also no information about the mental health of the respondents before the pandemic in this study. Therefore, it is not possible to discern the direct effect of the pandemic from possible pre-existing mental health problems of the respondents. Further, the analyzed data originated during the first Czech national lockdown in the spring of 2020, at the time when the disease spread was well contained. The following waves of the pandemic were more severe for the Czech population (Our World in Data, 2022); thus, the findings of this study might not be applicable to the entire pandemic period. Another limitation would be that the data consists of self-reports of respondents, which can be influenced by social desirability, especially at the adolescent age (Camerini and Schulz, 2018). Owing to lower level of cognitive maturity (Mwamwenda, 1995), adolescents may over-report the socially desirable experiences and underreport the undesirable ones (Krumpal, 2013). In the present study, social desirability could have increased subjective positive evaluations of family relationships and decreased reporting of psychological symptoms describing bad mood or feeling low, especially in boys, who tend to deny the emotional distress and negative affect compared to girls (Koenig et al., 1994).

## Conclusion

Long-term isolation, including lockdowns and quarantines, may have a distressing effect on anyone experiencing it. During the spring 2020 Czech lockdown, the negative impact was more apparent in girls. Adolescents’ perceived level of stress was connected to their health and well-being. The strongest predictor of higher level of PSS was frequent feeling of loneliness. On the contrary, lower level of PSS was most associated with having someone to talk to. Such knowledge is important not only for the ongoing pandemic, but also for possible future disasters that could affect our everyday lives. As it is difficult to predict what effect protective pandemic measures will elicit on the mental health of adolescents in the long run, the impacts still need to be carefully monitored. Psychological coping strategies to prevent the consequences of social isolation and development of mental health problems should be promoted on individual, family and even community level. In the case of an ongoing pandemic situation, policymakers should be aware of the risks of social isolation for adolescents and not to prolong the duration of isolation beyond what is necessary. The restrictions should be implemented with special care and considerations, especially in regions with low-income families where financial difficulties and household overcrowding could be more substantial. The adolescents need help with developing healthy coping mechanisms to be resilient to the short-and long-term psychological effects of the pandemic. The authorities need to ensure that support services are widely accessible to young people and their families in order to prevent longer-term mental health impacts. Moreover, families and communities should be provided with specific measures which could help monitor adolescents’ psychological well-being and health, and thus prevent the development of mental problems.

## Data availability statement

Data are available on reasonable request from the last author of the study (petr.badura@upol.cz). Access to HBSC Lockdown 2020 study data corresponds with the international HBSC study rules and is more precisely described at: https://hbsc.cz/lockdown2020/ (in Czech).

## Ethics statement

The studies involving human participants were reviewed and approved by Ethics Committee of the Faculty of Physical Culture, Palacky University Olomouc. Written informed consent to participate in this study was provided by the participants’ legal guardian/next of kin.

## Author contributions

JF, RZ, PT, and PB: conceptualization. PB, JF, NK, and DS: methodology. NK, DS, and PB: validation. JF: formal analysis, software, and visualization. RZ, PT, and PB: resources and funding acquisition. PB and JF: data curation. JF, NK, and PB: writing – original draft preparation. JF, NK, DS, RZ, PT, and PB: writing – review and editing. NK and PB: supervision. All authors contributed to manuscript revision, read, and approved the submitted version.

## Funding

This study was supported by the Czech Science Foundation (GACR), grant number 20-25019S; and by an internal grant agency of the Palacky University Olomouc, grant number DSGC-2021-0122.

## Conflict of interest

The authors declare that the research was conducted in the absence of any commercial or financial relationships that could be construed as a potential conflict of interest.

## Publisher’s note

All claims expressed in this article are solely those of the authors and do not necessarily represent those of their affiliated organizations, or those of the publisher, the editors and the reviewers. Any product that may be evaluated in this article, or claim that may be made by its manufacturer, is not guaranteed or endorsed by the publisher.

## Footnotes

^1^ http://www.hbsc.org
