# Supplementary material for: Perceived stress of adolescents during the COVID-19 lockdown: Bayesian multilevel modeling of the Czech HBSC lockdown survey
Source: Front Psychol. 2022 Sep 29;13:964313. doi: 10.3389/fpsyg.2022.964313 (PMC9558731; doi:10.3389/fpsyg.2022.964313)
Supplement: Supplementary file 1 [file Table_1.DOCX]

library(rethinking)

library(BayesianFirstAid)

#DATA LIST

data_list <- list(

PSS = d$zPSS,

lifesat = d$zLifesat,

PsyCom = d$zPsyCom,

SomCom = d$zSomCom,

health = d$zHealth,

region = d$kraj,

gender = d$gender,

age = d$zage,

grade = d$grade,

lonely = d$zLonely,

partgroup = d$zPartgroup,

talkto = d$zTalkto,

CmpSch = d$zCmpSch,

CmpLei = d$zCmpLei,

sleepSCH = d$zSleepSCH,

sleepWKD = d$zSleepWKD,

physact = d$zPhysact,

lostjob = d$lostjob,

workless = d$workless,

cramped = d$cramped,

learnnew = d$learnnew,

worries = d$worries,

disputes = d$disputes,

moretime = d$moretime

)

#----------------------------------

#CORRELATES OF PSS

bayes.cor.test(d$zPSS, d$zLifesat)

bayes.cor.test(d$zPSS, d$zPsyCom)

bayes.cor.test(d$zPSS, d$zSomCom)

bayes.cor.test(d$zPSS, d$zHealth)

#----------------------------------

#MULTILEVEL REGRESSION

#MODEL 1

m1 <- map2stan(

alist(

PSS ~ dnorm(mu,sigma),

mu <- a_region[region] + b1*lonely + b2*partgroup + b3*talkto +b4*gender +b5*age,

a_region[region] ~ dnorm(a,tau),

a ~ dnorm(0,1),

c(b1, b2, b3, b4, b5) ~ dnorm(0,1),

sigma ~ dcauchy(0,2.5),

tau ~ dcauchy(0,2.5)

), data=data_list, iter=1e4 , chains=4)

precis(m1, prob=0.95)

#----------------------------------

#MODEL 2

m2 <- map2stan(

alist(

PSS ~ dnorm(mu,sigma),

mu <- a_region[region] + b1*zCmpSch + b2*zCmpLei

+ b3*zsleepSCH + b4*zsleepWKD + b5*zphysact +

+ b6*gender + b7*age,

a_region[region] ~ dnorm(a,tau),

a ~ dnorm(0,1),

c(b1, b2, b3, b4, b5, b6, b7) ~ dnorm(0,1),

sigma ~ dcauchy(0,2.5),

tau ~ dcauchy(0,2.5)

), data=data_list, iter=1e4 , chains=4)

precis(m2, prob=0.95)

#----------

#COMPARE MODEL 1 MODEL 2 FIT

compare(m1, m2)

#----------------------------------

#MODEL 3

m3 <- map2stan(

alist(

PSS ~ dnorm(mu,sigma),

mu <- a_region[region] + b1*lostjob + b2*lessmoney + b3*cramped + b4*learnnew

+ b5*worries + b6*disputes + b7*moretime

+ b8*gender + b9*age,

a_region[region] ~ dnorm(a,tau),

a ~ dnorm(0,1),

c(b1, b2, b3, b4, b5, b6, b7, b8, b9) ~ dnorm(0,1),

sigma ~ dcauchy(0,2.5),

tau ~ dcauchy(0,2.5)

), data=data_list, iter=1e4 , chains=4)

precis(m3, prob=0.95)

plot(precis(m3, prob=0.95))

#----------

#COMPARE MODEL 2 MODEL 3 FIT

compare(m2, m3)
